# An Automated Visual Psychophysics Method to Measure Visual Function in Swine Preclinical Animal Model

**DOI:** 10.1167/tvst.13.3.8

**Published:** 2024-03-12

**Authors:** Francesca Barone, Irina Bunea, Kristi Creel, Ruchi Sharma, Juan Amaral, Arvydas Maminishkis, Kapil Bharti

**Affiliations:** 1 Ocular and Stem Cell Translational Research Section, National Eye Institute, NIH, Bethesda, MD, USA; 2 Translational Research Core, Ophthalmic Genetics and Visual Function Branch, National Eye Institute, NIH, Bethesda, MD, USA

**Keywords:** visual psychophysics, contrast sensitivity, visual acuity, large animal models, preclinical animal testing, cell therapy, gene therapy

## Abstract

**Purpose:**

The aim of this study was to develop and validate a test to assess visual function in pigs using the visual psychophysics contrast sensitivity function.

**Methods:**

We utilized a touchscreen along with a pellet reward dispenser to train three Göttingen pigs on a visual psychophysics test and determined their contrast sensitivity function. Images with different contrast resolutions were used as visual stimuli and presented against a control image in a two-choice test. Following animals’ acclimatization and the first phase of training, the system was arranged such that animals could self-run multiple consecutive trials without human intervention.

**Results:**

All animals were trained within a week and remembered the task with 1 day of reinforcement when tested 1 month after the last visual assessment. All trained animals performed well during the trial with minimal screen side bias, especially at contrast threshold above 40%.

**Conclusions:**

Göttingen pigs are trainable for a visual psychophysics test and able to self-run the trial without human intervention.

**Translational Relevance:**

Contrast sensitivity is one of the key parameters to assess visual function in humans. The possibility of measuring the same parameters in a large animal model allows for a better translation and understanding of drug safety and efficacy in preclinical ophthalmology.

## Introduction

Preclinical animal models and assessment of animal performance are critical tools required for the development of new treatments. In the eye, retina function and health are measured using imaging and functional modalities like optical coherence tomography, adaptive optics, and electroretinograms.^1^ Longitudinal analysis using these modalities provides information on retina health under diseased conditions and under treatment paradigms. However, all these modalities measure local retina health and do not provide information about functional vision of an animal.^2^^,^^3^ Functional vision is attained by the transmission of synaptic signals from retinal neurons to the visual cortex and correct processing of these visual stimuli in the brain, and it is measured by an individual's ability to successfully interact with its environment.^2^ The need to test functional vision is particularly critical for regenerative approaches such as cell and gene therapies that may help regenerate synaptic connections in the retina and perhaps all the way to the brain.^4^^–^^6^

In humans, multiple tests exist for functional vision measurement.^7^^–^^9^ These include microperimetry, best-corrected visual acuity (BCVA), contrast sensitivity (CS), reading speed, and visual-guided mobility tests.^7^^–^^9^ To correctly extrapolate results of preclinical testing of a drug from animals to humans, it is important that tests of functional vision are also performed as part of preclinical animal studies. However, some of the routine visual acuity-based tests that require structured linguistic abilities such as BCVA and reading speed are not feasible in animals. Some of the more frequently used functional vision tests for rodents include visual water test, optomotor reflex test, pattern electroretinography, and pattern visual evoked potentials.^10^ Most of these tests, however, measure what is called the pattern vision—an ability of our visual system to recognize stimuli arranged in a certain pattern and do not provide a complete picture of functional vision.^11^ Furthermore, some of these tests require sedation in large animals, such as nonhuman primates, swine, or canines, or are not feasible or reliable because of animal size and their ability to use nonvisual cues, such as smell, for navigation.^12^^,^^13^ Previously, tests have been developed to determine operant-based contrast sensitivity and visual texture modulation in monkeys, as well as visual acuity of pigs at different light intensities.^14^^–^^16^ More recently, significant success has been made using a visually guided navigation test of large animals for the assessment of cell and gene therapies.^17^^,^^18^ In fact, some of this work led to the development of the first US Food and Drug Administration (FDA)–approved gene therapy for a deficiency of RPE65 leading to childhood-onset blindness, underscoring the importance of developing such tests.^3^ However, one challenge with a visually guided navigation test is the confounding issue of animals’ ability to use non-vision-related olfactory abilities for navigation. It requires additional evaluation of parameters such as position of animals’ head during test performance. In any case, this is a powerful test for functional vision in large animals but does not allow for discrimination of fine changes in vision.

Contrast sensitivity is a test for functional vision that measures the ability of an individual to discern outlines of objects relative to their background, and CS function is obtained by measuring contrast thresholds over a range of spatial frequencies.^19^ It provides complementary information to the visual acuity (VA) tests since most VA tests are mostly performed under high-contrast conditions and do not measure an individual's CS function. It is thought that reduced CS can be seen even before a measurable drop in VA and, hence, provides a good indication of visual health of an individual and is associated with reduced quality of life.^20^

In this study, we developed a protocol for measuring CS function as a parameter of visual function in pigs. Göttingen minipigs were trained to recognize a black and white striped target image on a touchscreen display, compared to a fully gray image. The training was based on positive reinforcement, and animals were compensated with a reward (“treat”) when making nose contact with the target image. After the initial training, animals were presented with a different set of images: a control image (gray) and 10 striped target images with contrast ranging from 10% to 100%. Animals’ behavior was recorded using video cameras, and multiple parameters such as number of trials, bias toward one side of the screen, and latency in response timing were recorded on the computer connected to the touchscreen display. Our protocol shows that swine can be trained on CS tests using a touchscreen on a two-image discrimination test and remember their training for at least 1 month. By implementing our protocol with different threshold images, this test can be used to measure CS function and provides a reliable tool (at least for high-contrast levels) to assess changes in vision in large preclinical models.

## Methods

### Animals

Three healthy Göttingen minipigs (one female and two castrated males) between 3 and 4 years of age with normal vision and motor functions (Supplementary Fig. S1) were enrolled in the study. All animals in this study were housed and treated according to the National Institutes of Health and Association for Research in Vision and Ophthalmology guidelines for the humane treatment of animals in visual research. Animals were singly housed in large indoor enclosures (“pens”—approximately 6 feet by 10 feet) with elevated flooring and automated water dispensers and fed twice per day. Each pen had a covered outdoor area with cement flooring where animals were allowed to venture twice a day. All trials were performed in the hallway outside the animals’ pens for ease and familiarity of animals with the space, but it is possible to set up trials in any location in an enclosure with minimum size of 6 × 6 feet. Within the trial area, electric and connecting cables were taped to the wall or the floor. The pellet dispenser was physically secured by one of the operators; it is possible to mount the pellet dispenser to a wall. Animals were tested up to an hour at a time and not more than 3 days per week. No food restriction protocols were used. Food was administered two times a day, at 6:30 AM and at 2 PM. On the day of the trial the second meal was administered after the trial and not later than 4 PM. Water was offered ad libitum but not during the trial. During the trial, pellet treats (certified Supreme mini treats; Bio-serv, Flemington, NJ, USA) were automatically dispensed for every positive response. Multiple treat flavors were tried; animals preferred the very berry flavor. Commercial peanut butter used for human consumption was used in the first part of the training, to guide the animals to the touchscreen display.

### Apparatus

**Figure 1.**
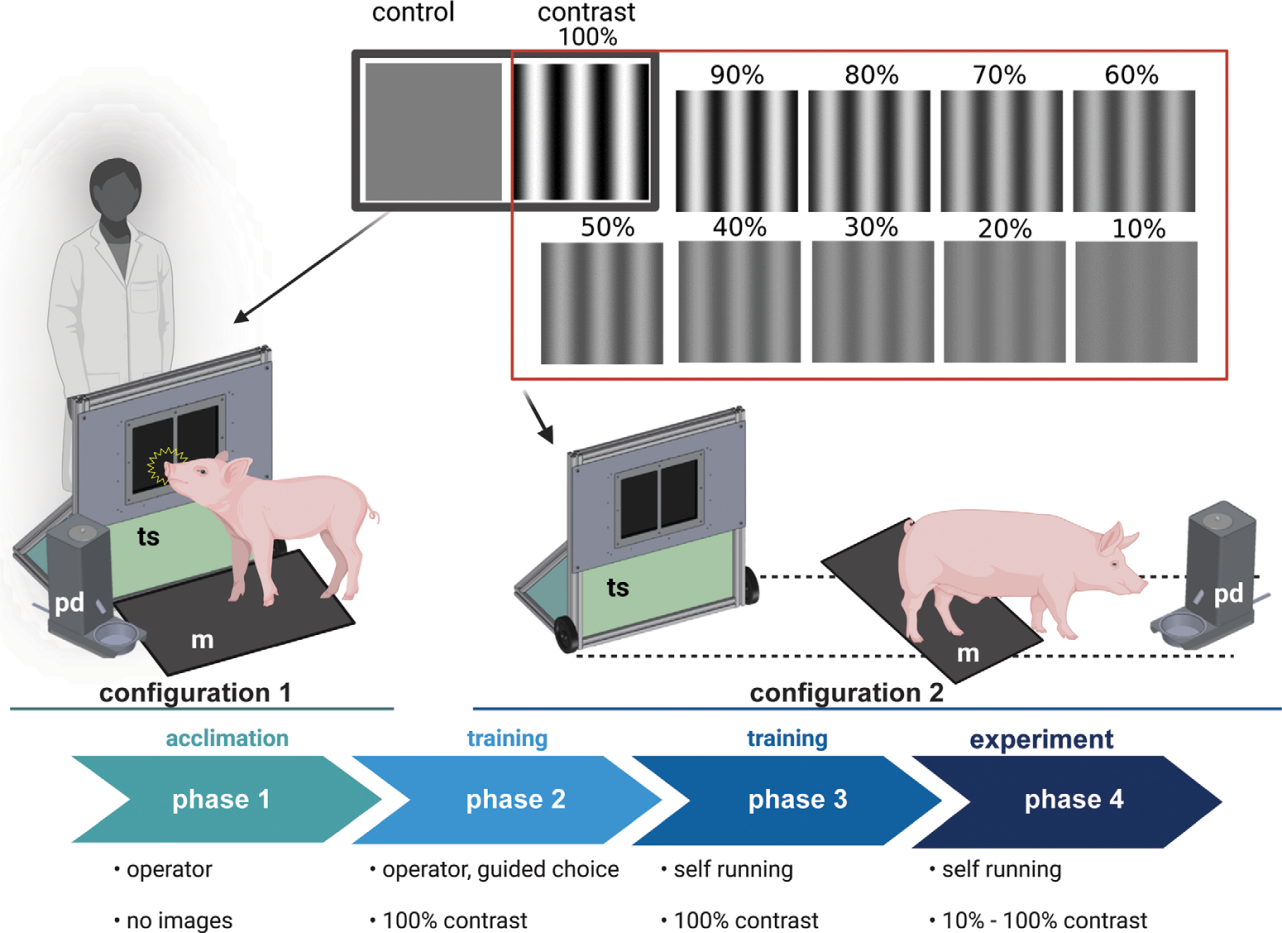
Study design and apparatus configuration during training and experimental phases. Phase 1—acclimation: a customized touchscreen (ts), a weight-sensitive mat (m), and a pellet dispenser (pd) are placed next to one another to allow the animal to acclimate to the apparatus and the enclosure. Phase 2—operator-guided training: a control gray image is presented against a 100% contrast image (*top left two panels*) and the animal is guided by the operator to touch the correct side of the screen. Apparatus setup is maintained in configuration 1. Phase 3—self-run training: apparatus setup is changed to configuration 2. Animal walks over the mat (m) to activate the screen, and if it touches the 100% contrast image on the touchscreen (ts), a pellet reward is dispensed in pd. Phase 4—experiment: touchscreen randomly displays 10 different images ranging in contrast from 10% to 100% stripes (images shown in the *top panel*) against a 100% gray image on the other side. Touchscreen gets activated by animal stepping over the mat. If the animal touches the correct side (striped side) of the screen, the pellet dispenser dispenses a reward. If the animal touches the incorrect side, no reward is dispensed.

A customized stand-alone CANTAB touchscreen (Lafayette Instrument, Lafayette, IN, USA) with adjustable height (the screen is 2 feet above the ground) was used. This customized design was modified from CANTAB touchscreen used for monkeys to better accommodate settings for Göttingen pig height, mobility, and trainability (apparatus design details provided in Supplementary Figs. S2 and S3). An infrared-based touchscreen equipped with a mask to divide the screen into two equal panels (left panel—side 1 and right panel—side 2) was used. For training, a gray control image was presented against an image with 100% contrast black and white stripes. At the time of trial, a gray control image was presented against 10 images with contrast ranging from 10% to 100% (Fig. 1). The system was equipped with an automated pellet dispenser (pd) and a weight-triggered trial start mat (m) (Fig. 1). The minimum threshold surface area of the touchscreen was set to 1 cm^2^ to allow for the animals’ snout to trigger a response. The touchscreen, the pellet dispenser, and the mat were organized with different configurations during the training and the trial phases (Fig. 1).

### Visual Psychophysics Training and Trial Protocol

The visual psychophysics (VP) test—contrast sensitivity—was based on the ability of an animal to recognize a target black/white striped image against a gray image, both presented with the same luminosity. Animals triggered the touchscreen using their snout to recognize the correct image (Fig. 1). The test was designed to present a new image when the animal stepped on the weight-triggered mat. If the animal correctly identified the target image, a treat was automatically released by a pellet dispenser in a metal bowl and the trial ended. If the control image (100% gray) was identified, the trial ended without a treat being dispensed. A new trial started with the activation of the weight-triggered mat. The mat, touchscreen, and the pellet dispenser were all situated in proximity in an operator-dependent configuration (configuration 1; Fig. 1) during the acclimation phase and phase 1. After the training, the mat, touchscreen, and the pellet dispenser were organized in a linear sequence with the mat in between the touchscreen and the pellet dispenser (configuration 2; Fig. 1). Swine VP training and trials were performed in four distinct phases (Fig. 1).

1. **Acclimation (Phase 1):** In the first phase, animals were allowed to familiarize themselves with the VP apparatus in configuration 1 for up to 3 days. During this acclimation period, the system was turned off (no stimulus provided to the animal), and the animals were able to freely express their natural explorative behavior. This allowed the trainer to identify hazardous situations (move connecting wires to the wall out of the animal's reach, find optimal space between system's parts—to limit pig's distractions within the enclosure).
2. **Operator-Dependent Training (Phase 2):** After acclimation, the system was turned on and only the 100% contrast image was presented against the gray control image. Configuration 1 was maintained to allow the operator to guide the animal toward the side of the screen presenting the target image and allow the association between making contact with it and the treat release from the pellet dispenser. To guide the animal during this initial training phase, operators used “special” treats (peanut butter provided on a tongue depressor) and vocal positive reinforcement by tapping on the correct side of the screen (Supplementary Video S1). When the animal touched the correct target, a single pellet treat was automatically dispensed in the pellet dispenser. The animal was guided to the pellet dispenser by the operator.
3. **Self-Run Training (Phase 3):** Once the animal was trained to recognize the target image and to touch the screen, the apparatus configuration was changed to configuration 2 (self-run). The animal was trained to turn back from the pellet dispenser over the mat, initiating a new round and a new set of images presented on the screen. Some animals had the tendency to avoid stepping on the mat or stepping partially without triggering a new round of the trial. To avoid this problem, the space around the mat was reduced by barriers, to ensure the animal could not avoid the mat. This training phase was self-run as the configuration of the system allowed for the animals to complete a trial and trigger a new trial without the intervention of an operator (Supplementary Video S2). Each training session consisted of 50 to 100 trials where the 100% contrast image or a control gray image was randomly presented on one side of the touchscreen. Results of this training were recorded to monitor animals’ progress over a 1- to 2-month training period. If an animal had >75% correct response with 100% contrast image, the training was considered successful, and the animal was moved to the experimental phase.
4. **Experimental Phase (Phase 4):** The VP test was designed to self-run for multiple consecutive trials without human intervention using configuration 2. Ten images ranging from 10% to 100% contrast were randomly presented against the control gray image for 10 times and used to assess animal's CS function, between 50 and 100 trials depending upon animals’ cooperation (Supplementary Video S3).

Step sequence (Supplementary Fig. S4) was entered into the computer system of the touchscreen monitor to conduct and record the self-run training and experimental phase. Percentage of correct responses, number of touches per side of the screen, and latency between screen activation and screen touch were recorded.

## Results

### Visual Psychophysics Training Phases

#### Operator-Dependent Training (Phase 2)

None of the animals were distracted by the presence (smells and vocalization) of other animals in the pens near the testing area. All animals displayed a positive reaction to the training, and hence, it was considered a behavioral enrichment. Using treats is important in establishing a task path for animal to begin training. The number of treats received during the training or the trials did not interfere with the animals’ regular diet; hence, it was not necessary to adjust the feeding portions.

#### Self-Run Training (Phase 3)

All three animals successfully switched from operator-dependent training to self-run training. Animals were noticed to be distracted from training anywhere between 50 and 100 consecutive trials of screen touch; at that point, animals were taken back to their home pen.

**Figure 2.**
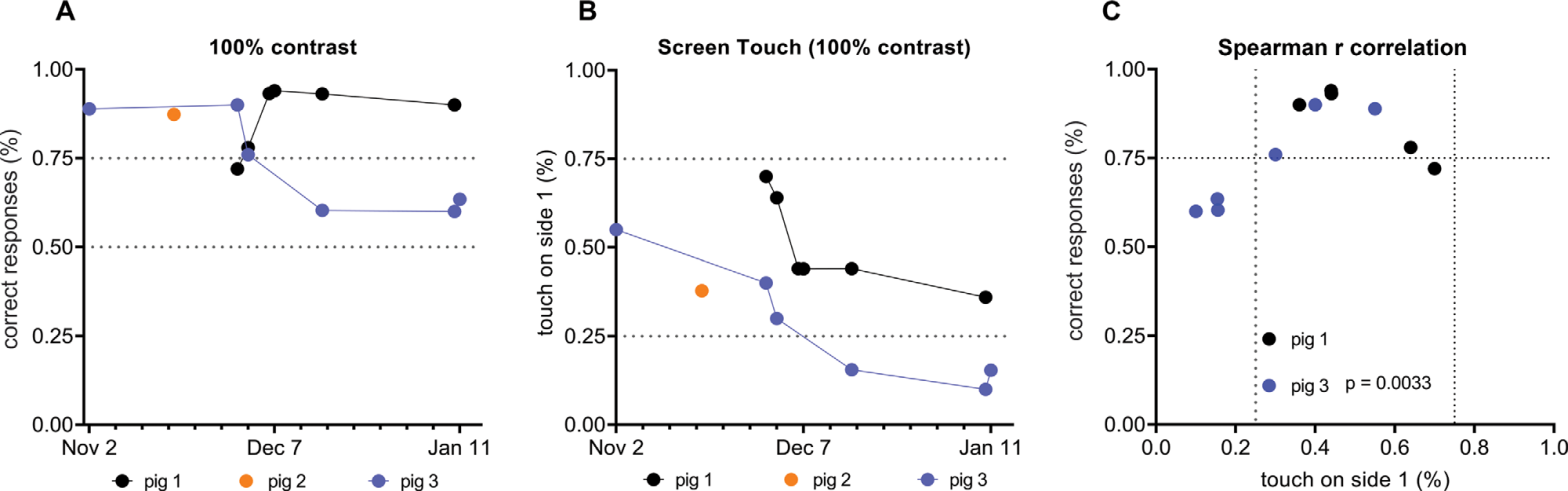
Self-run training results. (**A**) Percentage correct responses during the training period from November to January. Pig 2 training data were lost due to software malfunction. (**B**) Percentage of touches on one side of the screen. Touches closer to 50% suggest no bias, whereas touches less than 25% on one side (75% on the other) suggest significant screen side bias. (**C**) Spearman's correlation between correct response screen side bias for pigs 1 and 3.

While all animals were able to perform the task on their own, they had different learning curves. Overall, all animals were trained within a week as benchmarked by percentage correct answers ≥75% to identify a 100% contrast image against a control image (Fig. 2A). Pig 1 had six training sessions in which it improved from 70% correct responses to over 90% correct responses and remembered training for the 1-month period. In comparison, both pigs 2 and 3 started at around 90% correct response (follow-up training for pig 2 training could not be documented due to software problems). Similar to pig 1, pig 3 also remembered training for over a 1-month period, but it dropped in performance from 90% to over 60% over the 2-month period (Fig. 2A). Overall, two of the three pigs remembered their task for at least 1 month, suggesting partial success in training reinforcement. The number of touches on the same side of the screen was analyzed to evaluate if the animals preferred one of the screen sides (Fig. 2B). Since the correct image was randomly projected on either side of the screen, it had a chance of being 50% on one side of the screen. During training, when an animal picked correct responses, the animal touched the two screen sides approximately 50% of the time. This suggested there was no bias for a specific screen side. If animals’ performance dropped, it kept touching one specific side of the touchscreen, leading to screen bias. Pig 1, which maintained high correct response performance, also maintained nearly a 50% touch on each side of the screen, suggesting no side bias. For pig 2, lack of screen bias was maintained for the recorded training. However, pig 3, whose correct response performance dropped over time, also started to show bias toward one side of the screen and over 80% of the time picked one side of the screen. To better understand the correlation between correct responses and biased/unbiased touch on one side of the screen, we performed Spearman's correlation of these two data points (Fig. 2C). Our analysis confirmed that bias for one side of the screen was often associated with a wrong response.

#### Visual Psychophysics Test Experimental Phase (Phase 4)

**Figure 3.**
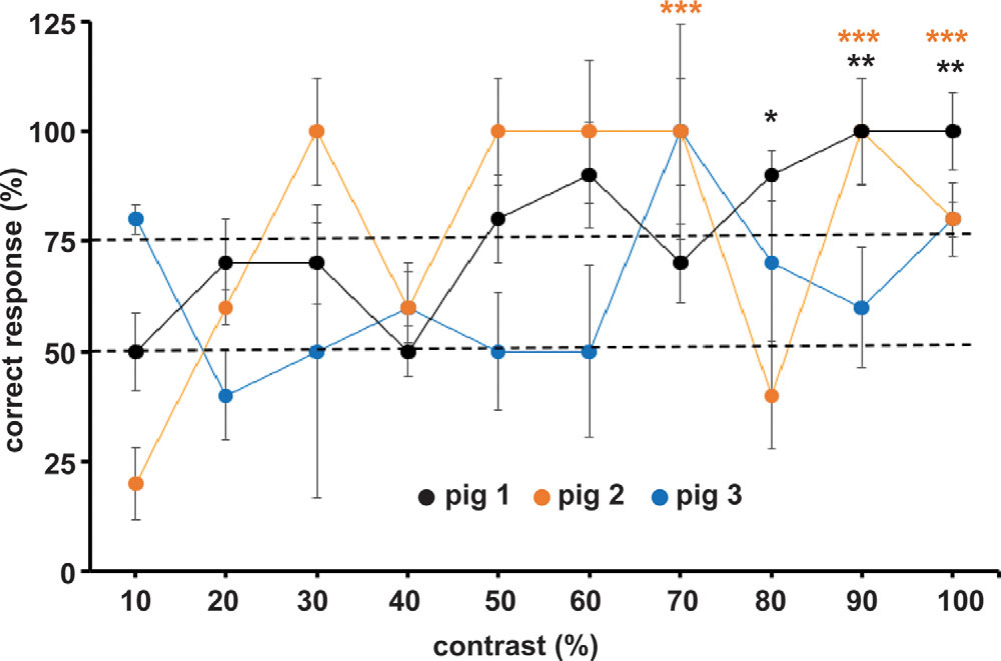
Experimental phase results. Graph shows percentage correct responses for all three pigs during the experimental phase where animals were shown 10 different images ranging from 10% to 100% contrast. Each animal performed at least four sessions, and each contrast level image was evaluated 10 times. For pigs 1 and 2, data points with more than 60% contrast showed statistical significance when compared to a random 50:50 chance event. *P < 0.05, **P < 0.01, ***P < 0.001, ns = nonsignificant. For pig 3, none of the data points showed any statistical significance.

In the experimental phase of this work, animals were shown images from 10% to 100% contrast. All images were randomly displayed on either side of the screen in no order. The ability of pigs 1 and 2 to pick the correct response started improving in images with contrast higher than 40%, with a sporadic drop in performance for images at 80% contrast for pig 1 and at 70% in pig 2. In contrast, pig 3 did not pick correct responses until the contrast threshold of 60%, and its responses did not show statistically significant differences from a random chance event, highlighting the individuality and the variability in animal training and performance (Fig. 3).

**Figure 4.**
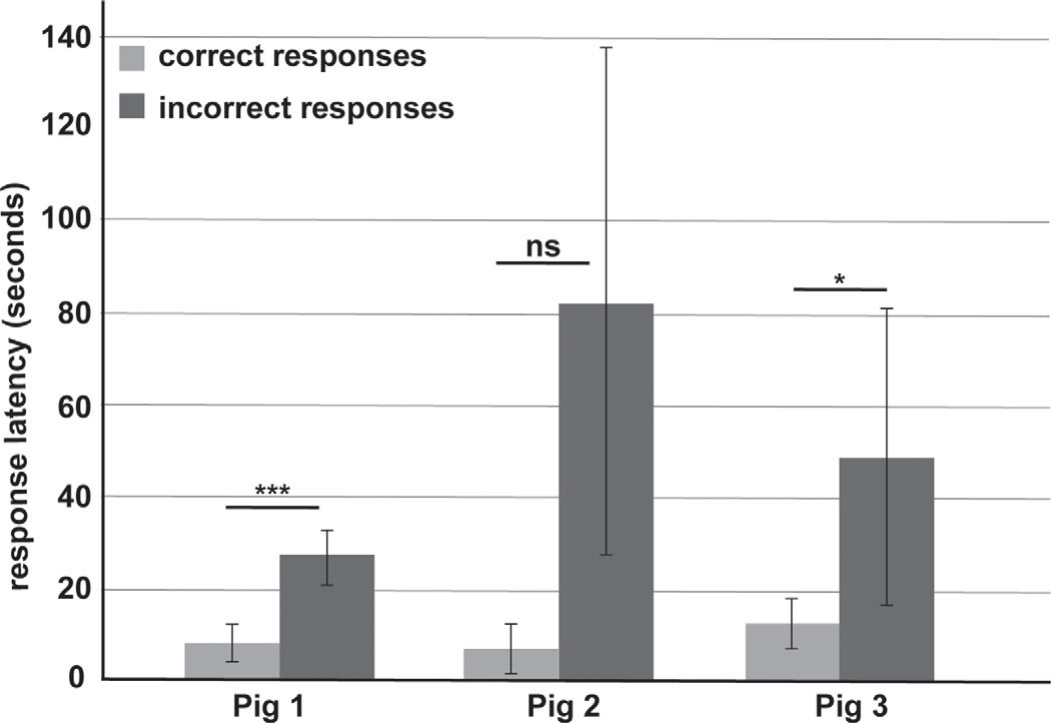
Response latency. Animals’ response time from screen activation to screen touch (latency) was calculated for all three pigs. Each animal performed at least four sessions, and each contrast level image was evaluated 10 times. *P < 0.05, ***P < 0.001, ns = nonsignificant.

To further analyze if an animal's ability to pick a correct response was related to their training, we analyzed their latency time—the time taken by an animal between screen activation and screen touch. The average latency time for pig 1 was 8.6 seconds for a correct response and three times longer for an incorrect response. In comparison, pig 2 took 7.3 seconds for a correct answer and almost 11 times longer for an incorrect response. Pig 3 took the longest for a correct response, taking almost 13 seconds, but similar to pig 1 took three times longer for an incorrect response (Fig. 4). Overall, a clear difference in latency time for picking a correct versus an incorrect response supported the notion that animals’ response was based on their training.

**Figure 5.**
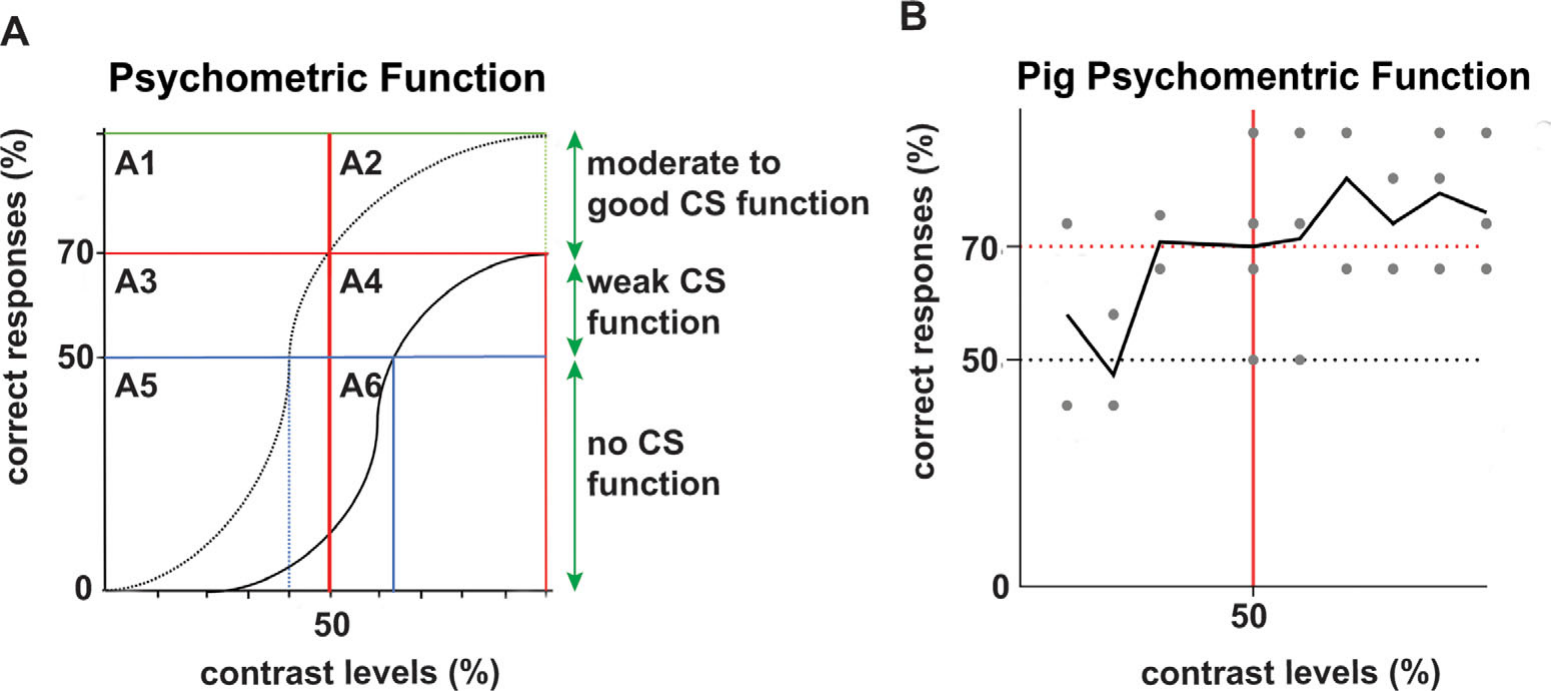
Psychometric function of animals. (**A**) Plots show six areas (A1–A6) for psychometric function or CS function evaluation. A1, good CS function; A2, moderate CS function; A3–A4, weak CS function; A5–A6, incorrect or randomly picked responses, weak training. (**B**) Twenty data points combined for all animals for response on images with contrast levels from 10% to 100% plotted against percentage of correct responses. Each animal performed at least four sessions, and each contrast level image was evaluated 10 times.

Lastly, we determined the contrast threshold using the visual psychometric function. The psychometric function is developed with the assumption that 100% of contrast is always recognized (100% correct response) and threshold is defined as the contrast level recognized 50% of the time. Figure 5A shows six areas (A1–A6): A1 shows animals are able to correctly identify images at low contrast, suggesting good CS function; A2 shows animals are able to correctly identify images at high contrast, with combined A1 and A2 data suggesting moderate to good CS function; A3 to A4 suggest most of the picked responses are still based on training, but animals have weak CS function because their correct response percentage was between 50% and 75%; and A5 to A6 show incorrect or randomly picked responses and suggest weak training. We plotted the data from all three animals based on this model (Fig. 5B). Twelve of 23 of our data points lie in the A1 and A2 quadrants, suggesting moderate to good CS function. Seven of 23 of our data points lie in A3 and A4, suggesting weak CS function. Four of our data points lie in A5 and A6, suggesting random selection. Overall, these data showed our training worked to detect CS function in these animals.

## Discussion

We developed a protocol to evaluate CS as a measure of VP function in pigs. This test uses a custom-built touchscreen system that randomly displays images with different contrast levels on one side of the screen against a gray tone (same luminosity) image on the other side. Our test expands on previously developed operant-based visual acuity tests for pigs that used different-sized Landolt-C symbols under different luminosity conditions to measure animals’ visual acuity.^16^ Different from the previous test, our test also measures animals’ bias toward one side of the screen and latency between screen activation and screen touch. Both these parameters can be used to determine if an animal has been well trained or needs additional training. Our data suggest that well training reduces screen side bias and latency time. In addition, the apparatus used in our test can also be used to measure contrast sensitivity under different luminosity conditions and allows varying frequency of stripes such that animals’ photopic and scotopic visual spatial frequency can be determined.

Animal training was performed on images with 100% contrast. To calculate animals’ contrast sensitivity and avoid biases with training images, the experimental phase utilized new images with contrast ranging from 10% to 100%. The 10% to 100% contrast image experiment confirmed the ability of our animals to detect images correctly, at least at contrast levels above 40%, allowing us to bin them according to their CS function and establish a baseline threshold for each animal. For two of the three animals used in our study, training worked well enough, allowing us to detect the range of their CS function. Furthermore, the CS function model we present here highlights different windows in which changes in animal CS function, from a baseline, can be measured in response to different therapeutic modalities.

Our protocol suggests several key features of a successful training: (1) animals need to be familiar and comfortable in their testing environment and free of unexpected stressors. To achieve this, we set up their training and trials right outside their pens in the hallway of our facility. Our apparatus is portable and can be used to set up training anywhere within an enclosed area of 6 × 6 feet. (2) Animals need to be comfortable with operators. We achieved this by having our operators spend time with animals on regular basis, including feeding, being in their pen, and positively interacting with the animals. (3) It is important to have animals be comfortable with multiple operators, adding redundancy in the need for a specific operator. (4) Animals need to acclimatize with the apparatus by freely expressing their natural explorative behavior. This was achieved by letting them walk around the test area for a couple of days in a row, without turning on any apparatus, and encouraging them with rewards (treats). (5) Connecting cables cannot be a distraction or safety concern for the animals. This was achieved by taping cables to the wall or the floor. (6) Separating the pellet dispenser from the touchscreen allowed animals to maneuver in the training area to retrieve the reward and then walk over the mat to initiate a new trial round, allowing for automation of the trial. The use of a weight-sensitive mat allowed animals to independently initiate new trials. Although not required for automation of this VP test, the screen activation trigger in the weight-sensitive mat only worked if animals had once stepped off (to retrieve the pellet) and stepped back on it. This ensured animals were oriented toward the touchscreen. This provided the possibility of determining latency in animals’ response as compared to a situation where the screen may automatically display a new image every few seconds, even if the animal was not ready. Our latency data clearly show that animals were confident and quicker in picking a correct response but took longer when picking an incorrect response.

Our experience suggests possible sources of errors for visual function testing: (1) more than a month of gap between animal training and the trial with no training reinforcement before the trial; (2) relative nearsightedness of pigs as compared to humans,^21^ leading to incorrect touch on the touchscreen if animals did not look it at a perpendicular angle; and (3) animal-to-animal variability in training and task performance. This variability suggests it is important to build a baseline for each animal when comparing an animal's behavior before and after the use of a therapeutic modality.

Each animal was able to perform 50 to 100 consecutive trials before fatigue was noticed and trials were stopped; all animals completed trials. However, each animal had a different baseline threshold for both the training and the experimental phase. Keeping in mind the individual behavior of each animal, we recommend this test be performed before and after a specific treatment, defining a baseline reading for each animal and evaluating changes upon the treatment. It is also important to note that all our trials were performed at ambient light conditions, suggesting that our tests only allowed measuring photopic visual function. This test is also doable under dim light conditions, which will allow measurement of an animal's scotopic vision as well. The images presented can be customized with colors to study different cone populations. In our case, animals were allowed to use both eyes for the CS test. But it is possible to patch one eye of the animal, allowing for testing of a therapy against the control in the same animal. One limitation of this method is that it requires prior training of animals. This may become limiting in cases where retina degenerates faster (e.g., in a swine retinitis pigmentosa model with Pro23His mutation where disease phenotype starts in 3-month-old animals).^22^ However, in cases of slower progressing diseases—such as in the case of a dog model of Stargardt disease,^23^ where disease phenotypes are not seen for years—this test may still be doable and may provide valuable insight into disease pathology. Even though we had trained and tested a relatively small number of animals, our data suggest that Göttingen pigs are trainable on a contrast sensitivity VP task.

Overall, our data provide evidence that swine (Göttingen strain) are trainable on a VP test, and this test can be used to discern the animals’ CS visual function. By refining the protocol and adding different spatial resolution, different luminosity, and color contrast images, it is possible to measure higher-resolution contrast sensitivity of the animals, a critical visual function feature in patients.^20^

## Conclusions

We developed a test to measure changes in contrast sensitivity visual function in awake animals. The test does not require long and/or repeated training and can be conducted without human interaction. The possibility of measuring the same parameters in humans and in large animal models allows for a better translation and understanding of drug efficacy in preclinical ophthalmology.

## Supplementary Material

Supplement 1

Supplement 2

Supplement 3

Supplement 4

Supplement 5

Supplement 6

Supplement 7
