# Supplementary material for: An Automated Visual Psychophysics Method to Measure Visual Function in Swine Preclinical Animal Model
Source: Transl Vis Sci Technol. 2024 Mar 12;13(3):8. doi: 10.1167/tvst.13.3.8 (PMC10941991; doi:10.1167/tvst.13.3.8)
Supplement: Supplement 1 [file tvst-13-3-8_s001.pdf]

Animal health records (before enrollment in the study)

Pig #1

Subjective: BAR, good attitude and appetite.
Temperature: 100.1
Heart Rate: 150
Respiration Rate: 16
Body Condition Score: 5/5
Mucous Membrane: pink and moist
CRT: Not performed
Hydration: No evidence of dehydration on visual cageside assessment
Eyes: No abnormal findings
Ears: Brown, waxy debris AU.
Oro-nasal: No abnormal findings.
Lymph Nodes: Not examined
Respiratory: No abnormal findings
Cardiovascular System: Not ausculted
Gastrointestinal: No abnormal findings
Urogenital: Not examined
Musculoskeletal: Normal ambulation. No lameness, non-painful
Nervous System: Normal mentation, no neurologic deficits noted.
Integument: No lesions noted.
Assessment: Apparently healthy animal
Plan: Semi-annual PE.

Pig #2

Subjective: BAR, good attitude and appetite.
Temperature: 100.0
Heart Rate: 128
Respiration Rate: 20
Body Condition Score: 5/5
Mucous Membrane: pink and moist
CRT: Not performed
Hydration: No evidence of dehydration on visual cageside assessment
Eyes: No abnormal findings
Ears: brown, waxy debris AU.
Oro-nasal: No abnormal findings.
Lymph Nodes: Not examined
Respiratory: No abnormal findings
Cardiovascular System: Not ausculted
Gastrointestinal: No abnormal findings
Urogenital: Not examined
Musculoskeletal: Normal ambulation. No lameness, non-painful
Nervous System: Normal mentation, no neurologic deficits noted.
Integument: Dry skin on vagina.
Assessment: Apparently healthy animal
Plan: Semi-annual exam

Pig #3

Subjective: BAR, good attitude and appetite.
Temperature: 100.7
Heart Rate: 130
Respiration Rate: 24
Body Condition Score: 5/5
Mucous Membrane: pink and moist
CRT: Not performed
Hydration: No evidence of dehydration on visual cageside assessment
Eyes: No abnormal findings
Ears: Brown, waxy debris AU
Oro-nasal: No abnormal findings.
Lymph Nodes: Not examined
Respiratory: No abnormal findings
Cardiovascular System: Not ausculted
Gastrointestinal: No abnormal findings
Urogenital: Not examined
Musculoskeletal: Normal ambulation. No lameness, non-painful
Nervous System: Normal mentation, no neurologic deficits noted.
Integument: No lesions noted.
Assessment: Apparently healthy animal
Plan: semi-annual PE

Animal health records (after study completion)

Pig#1

Subjective: BAR, good attitude and appetite. greasy, brown build up on skin. greasy debris in ears.
Temperature: 98.7
Heart Rate: 174
Respiration Rate: 28
Body Condition Score: 4
Mucous Membrane: pink and moist
CRT: Not performed
Hydration: No evidence of dehydration on visual cageside assessment
Eyes: No abnormal findings
Ears: No abnormal findings.
Oro-nasal: No abnormal findings.
Lymph Nodes: Not examined
Respiratory: No abnormal findings
Cardiovascular System: Not ausculted
Gastrointestinal: No abnormal findings
Urogenital: Not examined
Musculoskeletal: Normal ambulation. No lameness, non-painful
Nervous System: Normal mentation, no neurologic deficits noted.
Integument: No lesions noted.
Assessment: Apparently healthy animal
Plan: Complete acclimation procedures as routine, no additional follow-up necessary.

Pig #2

Hydration: No evidence of dehydration on visual cageside assessment
Eyes: No abnormal findings
Ears: No abnormal findings.
Oro-nasal: No abnormal findings.
Lymph Nodes: Not examined
Respiratory: No abnormal findings
Cardiovascular System: Not ausculted
Gastrointestinal: No abnormal findings
Urogenital: Not examined
Musculoskeletal: Normal ambulation. No lameness, non-painful
Nervous System: Normal mentation, no neurologic deficits noted.
Integument: No lesions noted.
Assessment: Apparently healthy animal
Plan: Complete acclimation procedures as routine, no additional follow-up necessary.

Pig #3

Subjective: BAR, good attitude and appetite. greasy build up on skin from sunscreen. degloved toenail growing back.
Temperature: unable to get.
Heart Rate: 124
Respiration Rate: 28
Body Condition Score: 4
Mucous Membrane: pink and moist
CRT: Not performed
Hydration: No evidence of dehydration on visual cageside assessment
Eyes: No abnormal findings
Ears: No abnormal findings.
Oro-nasal: No abnormal findings.
Lymph Nodes: Not examined
Respiratory: No abnormal findings
Cardiovascular System: Not ausculted
Gastrointestinal: No abnormal findings
Urogenital: Not examined
Musculoskeletal: Normal ambulation. No lameness, non-painful
Nervous System: Normal mentation, no neurologic deficits noted.
Integument: No lesions noted.
Assessment: Apparently healthy animal
Plan: Complete acclimation procedures as routine, no additional follow-up necessary.
