# Supplementary material for: An Automated Visual Psychophysics Method to Measure Visual Function in Swine Preclinical Animal Model
Source: Transl Vis Sci Technol. 2024 Mar 12;13(3):8. doi: 10.1167/tvst.13.3.8 (PMC10941991; doi:10.1167/tvst.13.3.8)
Supplement: Supplement 2 [file tvst-13-3-8_s002.pdf]

NO.	ECO	DESCRIPTION	BY	DATE

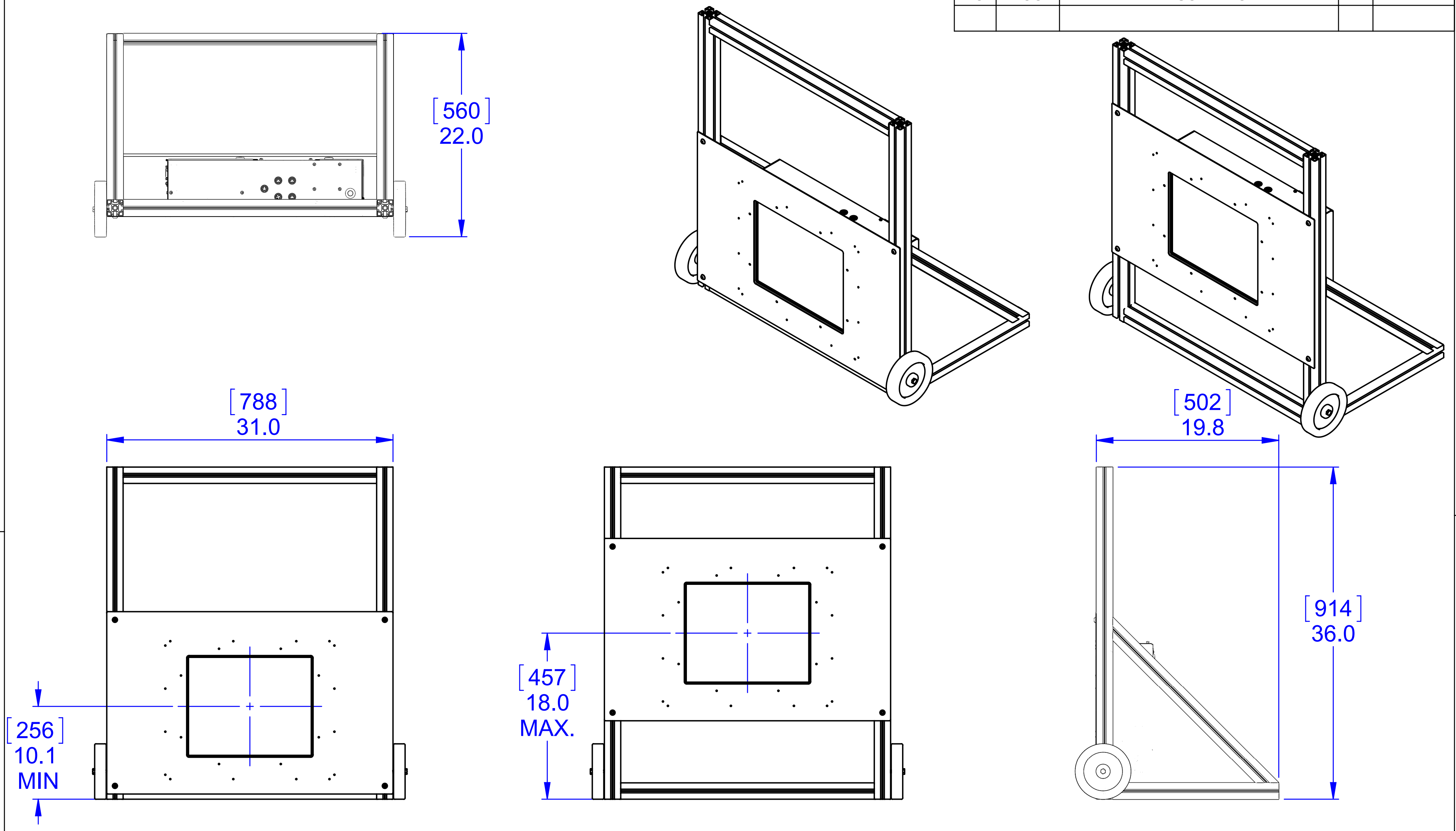

BREAK ALL CORNERS AND SHARP EDGES.

HANDLE PARTS WITH CARE. PARTS MUST BE FREE FROM SCRATCHES, BURRS, CHIPS, DIRT AND TOOL MARKS.

ALL ALUMINUM PARTS, STAINLESS STEEL AND PLASTIC SHEET MATERIAL MUST BE COATED WITH PROTECTIVE FILM. BLANKS MUST BE CUT WITH PROTECTIVE FILM ON INDICATED SIDE OF MATERIAL. PROTECTIVE FILM MUST BE LEFT IN PLACE UPON DELIVERY.

Ref. Prod. No.	TITLE<br>PIG CANTAB		
DO NOT SCALE PRINT<br>UNLESS OTHERWISE SPECIFIED	FINISH	DRAWN JMH	DATE 2/15/2019
MACHINE TOLERANCES<br>.XX = ± .010<br>.XXX = ± .005<br>ANGLE = ± .5°		CHECKED	DATE
	MATERIAL	LAFAYETTE INSTRUMENT CO.<br>3700 Sagamore Parkway, North<br>Lafayette, IN 47904<br>PH. 765-423-1505	FAX 765-423-4111
SHEET METAL TOLERANCES<br>.XX = ± .020<br>.XXX = ± .010<br>ANGLE = ± .5°	MATRL NO.		
THESE DRAWINGS AND SPECIFICATIONS ARE THE PROPERTY OF LAFAYETTE INSTRUMENT COMPANY AND SHALL NOT BE REPRODUCED, COPIED, OR USED AS THE BASIS FOR THE MANUFACTURING OR SALE OF AN APPARATUS OR DEVICE WITHOUT PERMISSION.		DWG. NO.	REV<br>0
		SHEET 1 OF 1	FORM SHEET - A
