# Supplementary material for: An Automated Visual Psychophysics Method to Measure Visual Function in Swine Preclinical Animal Model
Source: Transl Vis Sci Technol. 2024 Mar 12;13(3):8. doi: 10.1167/tvst.13.3.8 (PMC10941991; doi:10.1167/tvst.13.3.8)
Supplement: Supplement 3 [file tvst-13-3-8_s003.pdf]

NO.	ECO	DESCRIPTION	BY	DATE

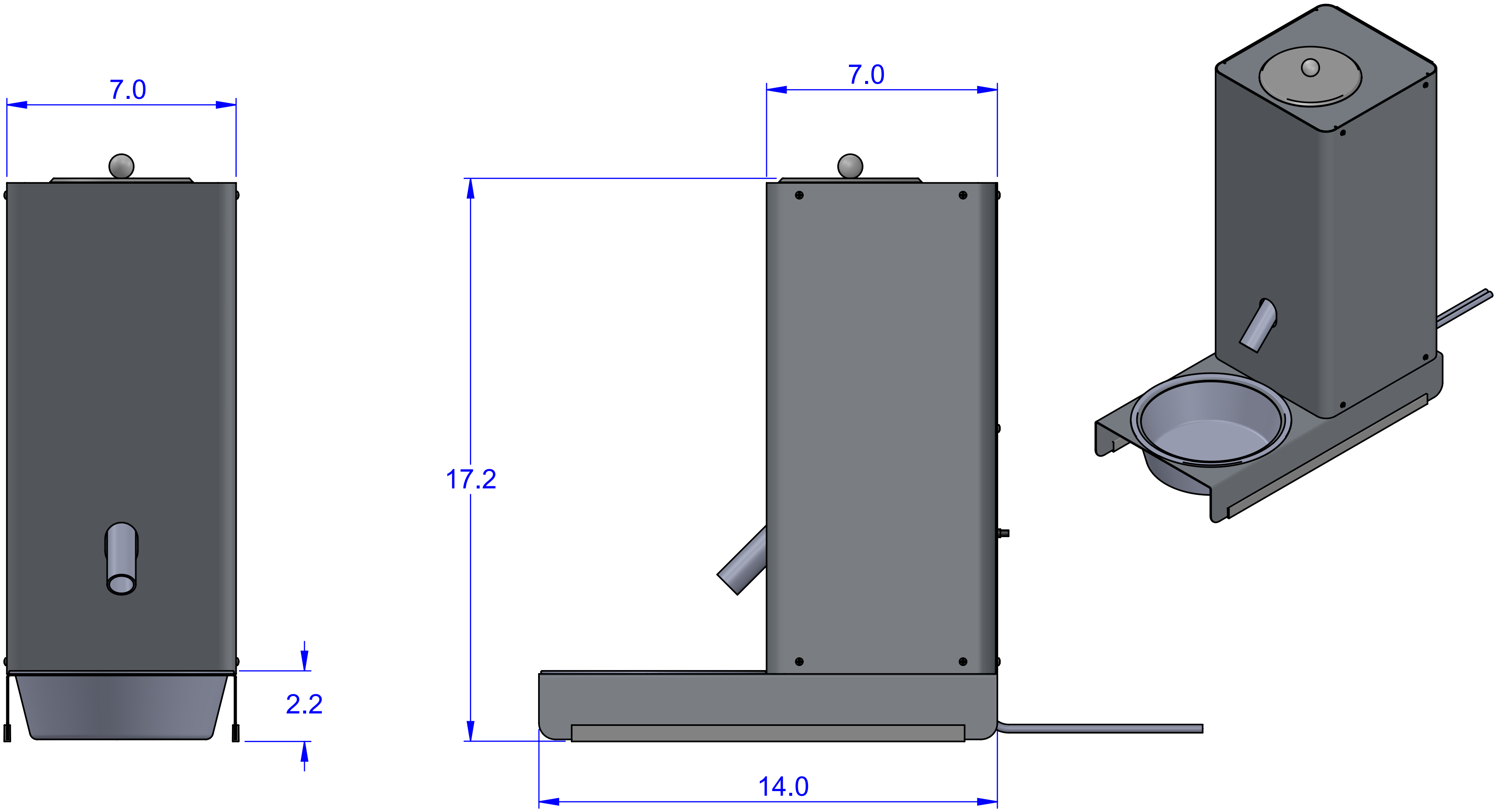

BREAK ALL CORNERS AND SHARP EDGES.

HANDLE PARTS WITH CARE. PARTS MUST BE FREE FROM SCRATCHES, BURRS, CHIPS, DIRT AND TOOL MARKS.

ALL ALUMINUM PARTS, STAINLESS STEEL AND PLASTIC SHEET MATERIAL MUST BE COATED WITH PROTECTIVE FILM. BLANKS MUST BE CUT WITH PROTECTIVE FILM ON INDICATED SIDE OF MATERIAL. PROTECTIVE FILM MUST BE LEFT IN PLACE UPON DELIVERY.

Ref. Prod. No.

TITLE
Pellet Dispensor

DO NOT SCALE PRINT
UNLESS OTHERWISE SPECIFIED

MACHINE TOLERANCES
.XX = ± .010
.XXX = ± .005
ANGLE = ± .5°

SHEET METAL TOLERANCES
.XX = ± .020
.XXX = ± .010
ANGLE = ± .5°

FINISH

MATERIAL
304 STAINLESS STEEL

MATRL NO.

DRAWN JMH | DATE 2/28/2019
CHECKED | DATE

LAFAYETTE INSTRUMENT CO.

3700 Sagamore Parkway, North
Lafayette, IN 47904

PH. 765-423-1505 FAX 765-423-4111

THESE DRAWINGS AND SPECIFICATIONS ARE THE PROPERTY OF LAFAYETTE INSTRUMENT COMPANY AND SHALL NOT BE REPRODUCED, COPIED, OR USED AS THE BASIS FOR THE MANUFACTURING OR SALE OF AN APPARATUS OR DEVICE WITHOUT PERMISSION.

DWG. NO. | REV

SHEET 1 OF 1 | FORM SHEET - A
