# Supplementary material for: An Automated Visual Psychophysics Method to Measure Visual Function in Swine Preclinical Animal Model
Source: Transl Vis Sci Technol. 2024 Mar 12;13(3):8. doi: 10.1167/tvst.13.3.8 (PMC10941991; doi:10.1167/tvst.13.3.8)
Supplement: Supplement 4 [file tvst-13-3-8_s004.pdf]

Schedule Name: training for pig

basic training with mat only. Gray as incorrect and image #10 (constrast 100%) as correct. No laser bean. Sound with incorrect, pellet with correct

Environment Link: Environment 1

Name: virtual chamber

Usage: 16 inputs/32 outputs

Location: Virtual Interface #1

Installed: Yes

Inputs		Outputs		Outputs with Intensity	
Line	Description/Label	Line	Description/Label	Line(s)	Description/Label

Conditions and Actions:

Group	Name	Goto	Condition	Action
1	Start - Correct=1	2	When **Correct_Position**.Value = 1	**Correct_Location** = **Correct_Position**.Value **Incorrect_Location** = 2
1	Start - Correct=2	2	When **Correct_Position**.Value = 2	**Correct_Location** = **Correct_Position**.Value **Incorrect_Location** = 1
2	Display Images	3	When switchmat #1 = Transition On	**Grey[Image 1]**.DisplayAtLocation (**Incorrect_Location**) **Image**.DisplayCurrentImageAtLocation (**Correct_Location**)
3	Incorrect Response	4	When **Grey**.GridTouch = **Incorrect_Location**	**Sounds**[Audio 1].PlayAudioForDuration, Duration= 500 ms, Intensity= 100 % **Image**.RemoveImageAtLocation (**Correct_Location**) **Grey**.RemoveImageAtLocation (**Incorrect_Location**) Increment **Incorrect_Counter** Start **ITI_Timer**
3	Correct Response	4	When **Image**.GridTouch = **Correct_Location**	Pulse pellet__dispenser #1 for 100 ms. Increment **Correct_Counter** **Image**.RemoveImageAtLocation (**Correct_Location**) **Grey**.RemoveImageAtLocation (**Incorrect_Location**) Start **ITI_Timer**
4	ITI End	1	When **ITI_Timer** >= 5	Increment **_Trial_Counter** GetNextImage **Image** Reset **ITI_Timer** GetNextValue **Correct_Position**
5	End of schedule.	-		No Action

Variables:

Name	Type	Value
_Schedule_Timer	Timer	0.000
_Trial_Counter	Integer	0
_Trial_Timer	Timer	0.000
Correct_Counter	Integer	0
Correct_Location	Integer	0
Incorrect_Counter	Integer	0
Incorrect_Location	Integer	0
ITI_Timer	Timer	0.000

Lists:

Correct_Position

Type: Integer

Mode: Random Equal Number

Values: 1, 1, 1, 1, 1, 2, 2, 2, 2, 2

Grey

Type: Image

Mode: Sequential

Grid Layout: Custom

Background Color: Black

Excluded Locations: N/A

Values: There is 1 image in this list, named "Image 1". Image previews are not available on reports.

Image

Type: Image

Mode: Random Equal Number

Grid Layout: Custom

Background Color: Black

Excluded Locations: N/A

Values: There is 1 image in this list, named "Image 10". Image previews are not available on reports.

Sounds

Type:

Mode: Sequential

Values:

Schedule Name: pig 10 contrast images= Gray as incorrect and image #10 (constrast 100%) as correct. No laser bean. Sound with incorrect, pellet with correct

Environment Link: Environment 1

Name: virtual chamber

Usage: 16 inputs/32 outputs=

Location: Virtual Interface #1=

Installed: Yes

Inputs		Outputs		Outputs with Intensity	
Line	Description/Label	Line	Description/Label	Line(s)	Description/Label

Conditions and Actions:

Group	Name	Goto	Condition	Action
1	Start - Correct=1	2	When **Correct_Position**.Value = 1	**Correct_Location** = **Correct_Position**.Value **Incorrect_Location** = 2
1	Start - Correct=2	2	When **Correct_Position**.Value = 2	**Correct_Location** = **Correct_Position**.Value **Incorrect_Location** = 1
2	Display Images	3	When switchmat #1 = Transition On	**Grey[Image 1]**.DisplayAtLocation (**Incorrect_Location**) **Image**.DisplayCurrentImageAtLocation (**Correct_Location**)
3	Incorrect Response	4	When **Grey**.GridTouch = **Incorrect_Location**	**Sounds**[Audio 1].PlayAudioForDuration, Duration= 500 ms, Intensity= 100 % **Image**.RemoveImageAtLocation (**Correct_Location**) **Grey**.RemoveImageAtLocation (**Incorrect_Location**) Increment **Incorrect_Counter** Start **ITI_Timer**
3	Correct Response	4	When **Image**.GridTouch = **Correct_Location**	Pulse pellet__dispenser #1 for 100 ms. Increment **Correct_Counter** **Image**.RemoveImageAtLocation (**Correct_Location**) **Grey**.RemoveImageAtLocation (**Incorrect_Location**) Start **ITI_Timer**
4	ITI End	1	When **ITI_Timer** >= 5	Increment **_Trial_Counter** GetNextImage **Image** Reset **ITI_Timer** GetNextValue **Correct_Position**
5	End of schedule.	-		No Action

Variables:

Name	Type	Value
_Schedule_Timer	Timer	0.000
_Trial_Counter	Integer	0
_Trial_Timer	Timer	0.000
Correct_Counter	Integer	0
Correct_Location	Integer	0
Incorrect_Counter	Integer	0
Incorrect_Location	Integer	0
ITI_Timer	Timer	0.000

Lists:

Correct_Position

Type: Integer

Mode: Random Equal Number

Values: 1, 1, 1, 1, 1, 2, 2, 2, 2, 2

Grey

Type: Image

Mode: Sequential

Grid Layout: Custom

Background Color: Black

Excluded Locations: N/A

Values: There is 1 image in this list, named "Image 1". Image previews are not available on reports.

Image

Type: Image

Mode: Random Equal Number

Grid Layout: Custom

Background Color: Black

Excluded Locations: N/A

Values: There are 10 images in this list. Image previews are not available on reports.
[1]: 'Image 10"
[2]: 'Image 2"
[3]: 'Image 3"
[4]: 'Image 4"
[5]: 'Image 5"
[6]: 'Image 6"
[7]: 'Image 7"
[8]: 'Image 8"
[9]: 'Image 9"
[10]: 'Image 10_1"

Sounds

Type:

Mode: Sequential

Values:
